# Management of Latent Tuberculosis Infection in Saudi Arabia: Knowledge and Perceptions Among Healthcare Workers

**DOI:** 10.7759/cureus.29134

**Published:** 2022-09-13

**Authors:** Hani S Almugti, Hussam M Alfaleh, Turki M Alshehri, Khaled Q Mokili, Abdul-Aziz M Al Qahtani, Hassan S Al Qahtani, Mohammed Z Alsayed, Mohammed A Al Asmari, Majed M Al Asiri, Mohammed A Al Amri, Ali F Al Fadhil, Bairam A Al Qahtani, Esmaeel S Al Bakrah, Humood A Shaikh, Mohammed G Al Shiq, Yahya A Al Shaik

**Affiliations:** 1 Medicine, Ministry of National Guard Health Affairs, King Abdullah International Medical Research Center, King Saud bin Abdul-Aziz University for Health Sciences, Jeddah, SAU; 2 Family Medicine, Prince Sultan Military Medical City, Riyadh, SAU; 3 Histopathology, Armed Forces Hospital Southern Region, Khamis Mushait, SAU; 4 Laboratory, Armed Forces Hospital Southern Region, Khamis Mushait, SAU; 5 Genetics, Armed Forces Hospital Southern Region, Khamis Mushait, SAU; 6 Pathology, Armed Forces Hospital Southern Region, Khamis Mushait, SAU; 7 Serology, Armed Forces Hospital Southern Region, Khamis Mushait, SAU; 8 Microbiology, Armed Forces Hospital Southern Region, Khamis Mushait, SAU; 9 Laboratory, Regional Laboratory and Central Blood Bank, Khamis Mushait, SAU; 10 Dentistry, Directorate of Health Affairs, Khamis Mushait, SAU

**Keywords:** latent tuberculosis, saudi arabia, treatment, knowledge, healthcare workers

## Abstract

Background

Tuberculosis (TB) continues to pose a serious threat to public health despite great efforts. For many years, management and screening for active TB cases have been the main focus of TB control programs. Latent TB is a stage where TB can be prevented and controlled. Therefore, designing a comprehensive TB control program that includes latent tuberculosis infection (LTBI) management diseases is needed to be implemented among the healthcare workers (HCWs) who have been found to be at a higher risk for active TB compared to the general population.

The objective of the study

The objective of the study is to assess the knowledge and perceptions of LTBI among HCWs. In addition to estimating the prevalence of LTBI among HCWs using closed-end questions in a self-administered questionnaire.

Subjects and methods

Through a cross-sectional study and non-random sampling technique, 324 (84%) healthcare workers who met the inclusion criteria completed and submitted the electronic questionnaire.

Results

Among all participants, the study reported a good knowledge about LTBI; however, a third of HCWs had poor knowledge about the difference between LTBI and active TB. Eighteen percent of participants were diagnosed with LTBI, and two-thirds accepted the treatment. Of all participants who started the treatment, 55% completed the treatment course. The compliance rate was high among young HCWs and physicians who had a short course of LTB treatment regimen.

Conclusion

The study reported a low acceptance and completion rate of LTBI therapy among HCWs. Low knowledge about some clinical facts of LTBI, the long duration of treatment, and being the treatment optional in Saudi health institutes were all barriers to accepting and completing the treatment of LTBI. All of these factors need to be addressed to increase the compliance rate to LTBI treatment.

## Introduction

With the absence of clinical manifestations, latent tuberculosis infection (LTBI) is defined as a state of the persistent immune response against antigens of a bacterium called *Mycobacterium tuberculosis (M. tuberculosis)* [1]. Globally, one-third of the population is estimated to have LTBI [1, 2]. Although there is no risk of spreading the infection to others, previous studies reported that 5-10% of LTBI patients would develop active tuberculosis (TB) disease, usually within the first five years after initial infection [2].

Clinically, LTBI used to be screened by utilizing either the tuberculin skin test (TST) or the interferon-gamma release assay (IGRA) [3]. IGRA has the advantage of being unaffected by bacillus Calmette-Guérin (BCG) vaccination [4], and several systematic reviews suggested that IGRAs are sensitive, specific, and more practical than TST in identifying LTBI, particularly in low TB-incidence settings [5].

Healthcare workers (HCWs) are more susceptible to TB than the general population [6, 7]. Saudi Arabia, throughout the previous ten years, had a low cumulative incidence rate of TB of 10 (8.7-12) per 100 000 population [1]. A report from a Saudi survey showed that the overall prevalence of LTBI among the general population was similar using TST and IGRA (9.3% and 9.1%, respectively) [8]. Compared with the general population, the prevalence rate of LTBI cases was higher (24%) among HCWs in a tertiary academic hospital in Riyadh [9].

Centers for Disease Control and Prevention (CDC) recommend TB screening programs for all HCWs and offer treatment for LTBI if diagnosed throughout the screening. In 2020, CDC and National Tuberculosis Controllers Association (NTCA) released updated guidelines on treating LTBI [10]. Adherence to the treatment course was a challenge in the success of the LTBI management program and an essential determinant of clinical benefit for the individual. Strategies to enhance adherence might involve using the short-course rifamycin-based LTBI treatment regimens that are considered safe, effective, and have higher completion rates than longer six to nine-month regimens [4]. As an effective alternative to LTBI treatment regimens, six or nine months of daily isoniazid (INH) is recommended if there are drug interactions with rifamycin [10].

Saudi Arabia is a country with high income and has a considerable labor force of HCWs from countries with high TB-endemic rates. Therefore, establishing a TB screening program in Saudi health care facilities will help discover and treat individuals with latent tuberculosis infection, thereby preventing infected individuals from developing active TB disease and stopping the spreading of TB to others. From healthcare workers' perception, the present study aims to enhance the ability of occupational health programs in Saudi healthcare facilities to screen and initiate treatment against LTBI.

## Materials and methods

Aim of the study

The aim of the study was to raise awareness about latent tuberculosis among healthcare workers in Saudi Arabia and enhance the ability of occupational health programs at healthcare facilities to screen and initiate preventive treatment against LTBI in order to reduce the incidence of active tuberculosis cases.

Primary (Specific) Objective

The primary objective was to assess the knowledge and perceptions of LTBI among HCWs, and to estimate the prevalence of LTBI among HCWs using closed-end questions in a self-administered questionnaire.

Secondary Objectives

The secondary objectives were to estimate the screening tool with high use rate in diagnosing LTBI in HCWs in Saudi health care facilities, measure the proportion of HCWs who started LTBI treatment, and to measure the proportion of HCWs who started LTBI treatment and completed the treatment course. 

This study was a cross-sectional study.

Study area/setting

The study was carried out in Saudi Arabia. The population of the current research were approached through the electronic questionnaire that was distributed on Facebook, Whatsapp, and Twitter, which are considered the most popular social media used by the Saudi nation.

Study subjects

The study recruited healthcare workers who met the inclusion criteria as the following: 1) employees who are working inside the healthcare facility and including but are not limited to: physicians, dentists, nurses, emergency medical personnel, laboratory technicians, pharmacists, and administrative staff. 2) Healthcare workers who are currently working at all the health institutes in Saudi Arabia of both gender and all nationalities.

Sample size and technique

Three hundred eighty-four healthcare providers out of 400,000 [11], the total number of healthcare providers who met the inclusion and exclusion criteria, were recruited in the present study with a confidence level of 95% and a 5% margin of error.

Through the snowballing sampling technique (non-probability sampling), the study recruited health care workers who met the inclusion and exclusion criteria by using a self-administered online questionnaire.

Data collection methods, instruments used, and measurements 

Variables of the Study

The dependent variables were the knowledge level of LTBI and the prevalence rate of LTBI, whereas the independent variables were age, gender, nationality, professions, clinical history, and hospital sector or type.

Knowledge Level of LTBI

In order to assess the level of knowledge regarding LTBI, an expert panel from family medicine, preventive medicine, occupational medicine, and internal medicine formulated six close-ended questions about the basic facts of LTBI. These questions were tested for validity by the expert panel, and the reliability was assessed through a pilot study. The Cronbach alpha coefficient was 0.8, indicating good internal consistency. 

Prevalence Rate of LTBI

Routinely, screening against TB is performed for the HCWs at the time of employment and then annually once employed. The subject was considered to have LTBI if he/she was on medications or a physician had diagnosed him with LTBI.

Questionnaire

The questionnaire of this study was self-administered and consisted of three sections. The first section included the demographic data (age, gender, nationality, profession, and hospital (governmental or private)). The second section included a knowledge assessment of LTBI, and the third section included 10 questions about the medical history of latent TB (Appendix 1).

Data Management and Analysis Plan

The Windows-based SPSS version 20 statistical software suite (IBM Inc., Armonk, New York) was utilized for data entry and statistical analysis. Data entry and coding stages were performed to enhance the data quality. For qualitative variables, data are presented using frequencies and percentages, and for quantitative variables, means and standard deviations were used. The Chi-square test and Fisher exact test were used to assess the association between participants' answers and other variables.

## Results

General demographic characteristics of participants

Of the study population, 324 (84%) subjects completed and submitted the questionnaire. Table 1 shows that half of the participants were under 30 years old, and almost 60% were Saudi HCWs from military hospitals.

**Table 1:** Demographic characteristics of participants (n=324)

Demographic characteristics	Frequency (n)	Percent (%)
Age category		
20-30 years	172	53
31-40 years	144	44
41-50 years	8	3
Gender		
Male	176	54
Female	148	46
Nationality		
Saudi	204	63
Non-Saudi	120	37
Health Sectors		
Governmental hospitals (Ministry Of Health)	92	28
Governmental mospitals (military)	112	65
University mospitals	8	3
King Faisal Specialist Hospital & Research Centre	8	3
Private hospital	4	1

**Figure 1:**
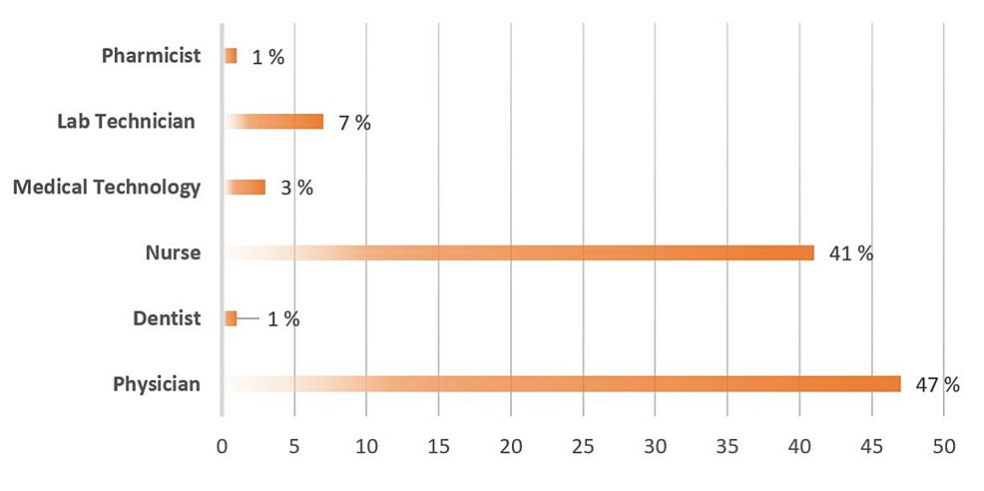
Percentages of participants according to their professions (n=324)

**Figure 2:**
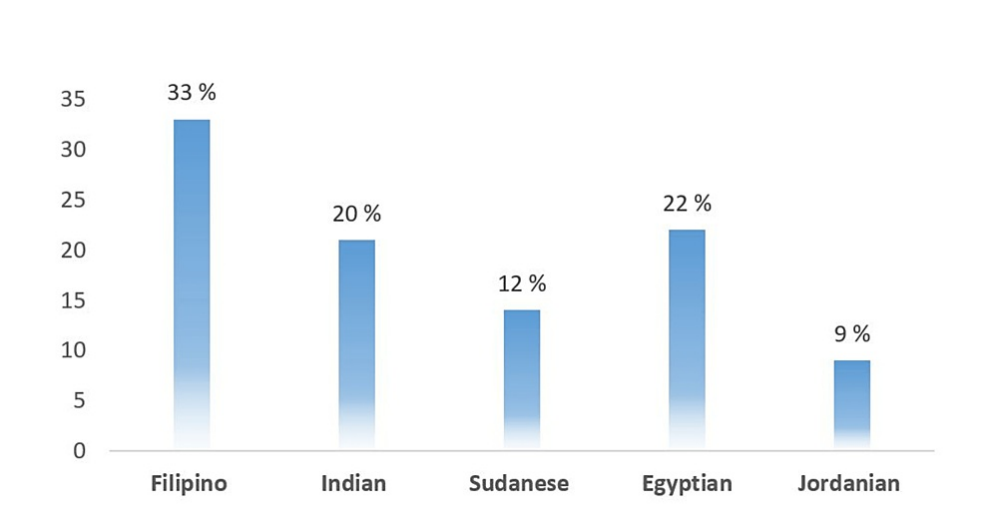
Nationalities of Non-Saudi participants (n=120)

Regarding the participants' profession, half were physicians, and 41% were nurse staff (Figure 1). The most commonly reported non-Saudi nationalities were Filipino, Indian, and Sudanese at 38%, 20%, and 12%, respectively (Figure 2).

Screening program for TB

**Figure 3:**
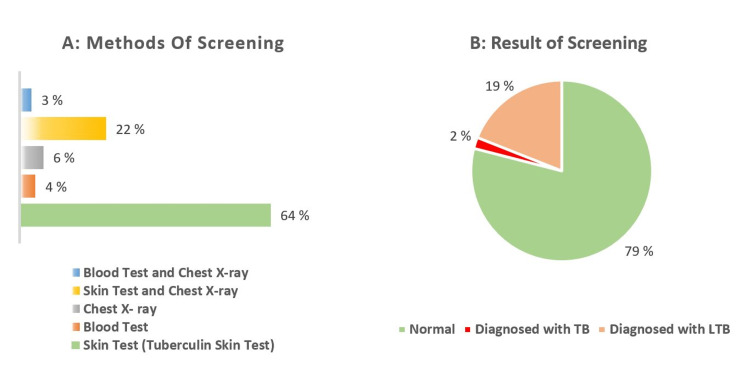
Medical tests used for TB screening (A) and the percentage of the discovered cases of TB or LTB (B) (n=324) TB - tuberculosis, LTB - latent tuberculosis

Several tools were used for screening; most participants had the TB skin test annually, and only 4% underwent TB blood test screening (Figure 3A). Among 324 HCWs in the present study, 60 (19%) were diagnosed with LTBI (Figure 3B).

Participants' knowledge about LTBI

As shown in Table 2, the majority of participants answered correctly to the knowledge assessment questions. However, a third of them do not know the following facts: there are no clinical symptoms of LTBI, and LTBI cases can not spread the infection to others.

**Table 2:** Participants' knowledge about the LTBI (n=324) TB - tuberculosis, LTBI - latent tuberculosis infection, BCG - bacillus Calmette-Guérin

Questions of knowledge assessment	Correct Answer n(%)	Wrong answer n(%)
What are the main symptoms that indicate latent TB infection?	224 (69 %)	100 (31%)
Can latent TB infection be spread from person to person?	240 (74%)	84 (26%)
Can latent TB infection be treated with prescribed TB medicine?	288 (90%)	36 (11%)
What is the benefit of treating latent TB infection?	296 (91%)	28 (9%)
How long does the treatment of latent TB infection last?	268 (83%)	56 (17%)
Do you think the BCG vaccine (a vaccine for TB) completely protects you from TB or latent TB for your whole life?	276 (85%)	48 (15%)

Management plan and treatment experience from LTBI cases

**Figure 4:**
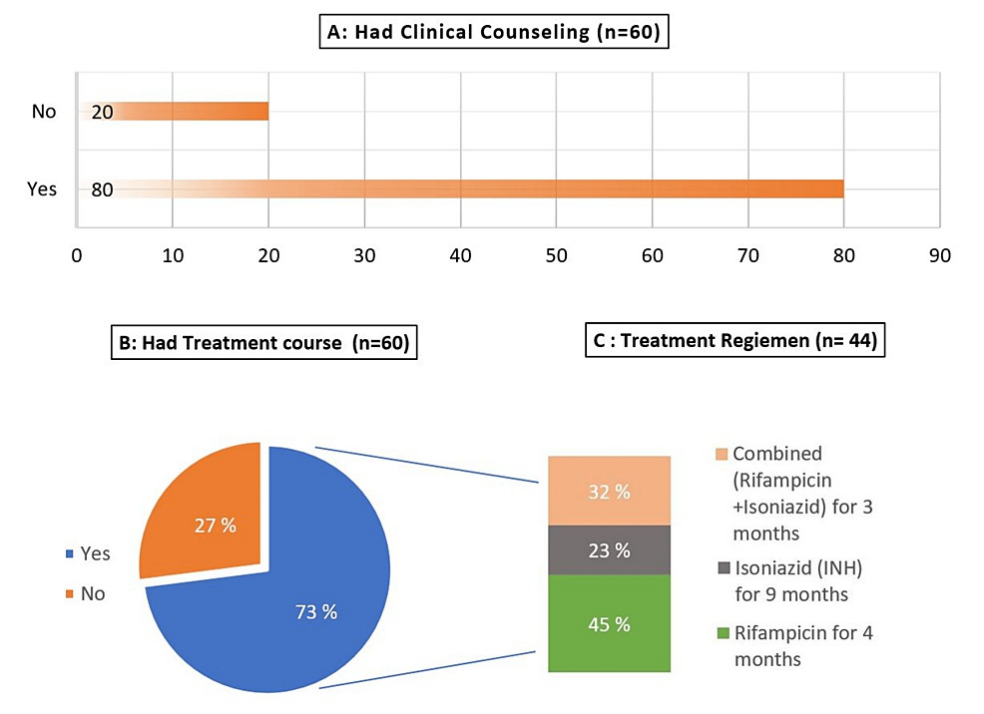
Management plan for the diagnosed cases of LTBI (n=60) (A) clinical counseling, (B) percentage of LTBI cases who started treatment, and (C) type of treatment regimen that they began with LTBI - latent tuberculosis infection

According to the participants who had LTBI (Figure 4A), two third of them received clinical counseling about their health condition provided by the employee health clinic. However, about a third of them refused to start the treatment therapy for LTBI (Figure 4B),

From Figure 4C, we can see that four months of rifampicin is the regimen of choice among half of the LTBI cases in the present study, followed respectively by the combined regimen (rifampicin+isoniazid) for three months (32%) and isoniazid (INH) for nine months (23%).

**Figure 5:**
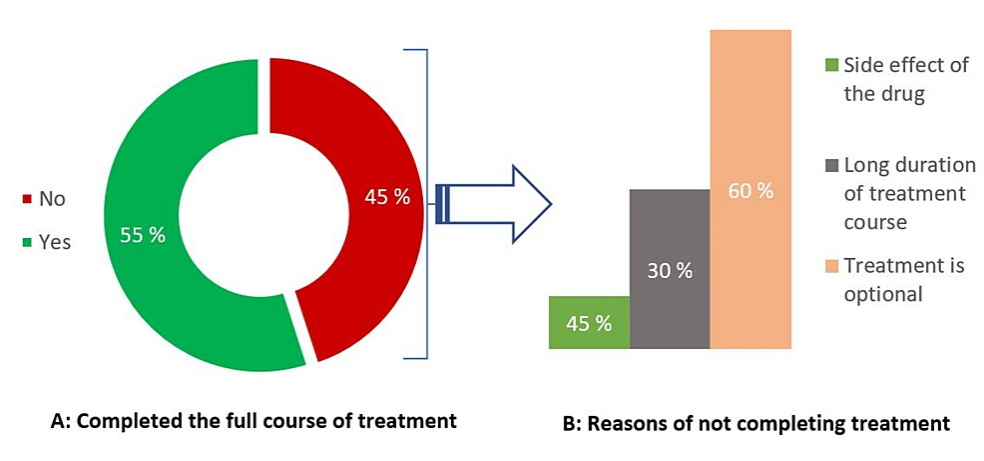
Percentage of LTBI cases who completed or did not complete the treatment course (A) and the common reasons behind not completing the treatment (n=44) LTBI - latent tuberculosis infection

The overall completion rate was 55%, and almost half of LTBI cases who started the treatment course reported low compliance with treatment. The majority (60%) stated that they stopped because it is an optional course of therapy, and a third of them complained of the long duration of treatment (Figure 5).

The results, as shown in Table 3, indicate that of all the participants of LTBI who completed the treatment course, 90% were on the short course treatment of the rifamycin-based regimen. On the other hand, 60% of non-completed LTBI treatment participants reported that the treatment being optional was the reason for their low compliance. 

**Table 3:** Regimens of LTBI treatment used by participants (n=44) LTBI - latent tuberculosis infection (*) Statistically significant at p<0.05

Treatment experience	Type of LTBI treatment regimen		
	Combined (rifampicin+isoniazid) for three months	Rifampicin for four months	Isoniazid (INH) for nine months
Completed the course of treatment			
No (n=20)	8	8	4
Yes (n=24)	10	12	2
p-value	0.001*		
Reasons for non-completed the treatment course			
Side effects (n=2)	2	0	0
Long duration of treatment (n= 6)	1	2	3
Treatment is optional (n =12)	2	7	3
p-value	0.001*		

Demographic and occupational characteristics of LTBI cases

Table 4 illustrates that two third of LTBI cases were female and less than 30 years old and half of them completed the treatment course for LTBI. A third of LTBI cases were Saudis. Moreover, the Filipino workers constituted a high percentage of cases among non-Saudis HCWs. Interestingly, two-thirds of Filipino LTBI cases completed the treatment course, and only 20% of Saudi patients reported the completion of treatment.

**Table 4:** Demographic and occupational characteristics of LTBI cases (n=60) LTBI - latent tuberculosis infection (*) Statistically significant at p<0.05

	Diagnosed with LTBI (n=324)		Had clinical counseling (n=60)		Started treatment course (n=60)		Started and completed the treatment course (n=44)	
	No (n)	Yes (n)	No (n)	Yes (n)	No (n)	Yes (n)	No (n)	Yes (n)
Age category								
20-30 years	132	40	8	32	8	32	12	20
31-40 years	128	16	4	12	4	12	8	4
41-50 years	4	4	0	4	4	0	0	0
p-value	0.01*		0.003*		0.001*		0.001*	
Gender								
Male	152	24	4	20	12	12	7	5
Female	112	36	8	28	4	32	13	19
p-value	0.02*		0.04*		0.001*		0.07	
Nationality								
Saudi	184	20	2	18	4	16	12	4
Filipino	25	15	3	12	3	12	1	11
Indian	17	8	3	5	3	5	0	5
Sudanese	12	5	1	4	2	3	0	3
Egyptian	18	9	2	7	3	6	5	1
Jordanian	8	3	1	2	1	2	2	0
p-value	0.6		0.023*		0.22		0.07	
Profession								
Physician	132	20	4	16	4	16	2	14
Dentist	4	0	0	0	0	0	0	0
Nurse	100	32	4	28	8	24	14	6
Medical technology	8	0	0	0	0	0	0	0
Lab technician	20	4	4	0	4	0	4	0
Pharmacist	0	4	0	4	0	4	0	4
p-value	0.001*		0.76		0.001*		0.001*	
Health sector								
Ministry Of Health	176	36	8	28	8	28	16	12
Military hospitals	76	16	4	12	8	8	3	5
King Faisal Specialist Hospital & Research Centre	0	8	0	8	0	8	1	7
University hospitals	8	0	0	0	0	0	0	0
Private hospitals	4	0	0	0	0	0	0	0
p-value	0.001*		0.007*		0.001*		0.008*	

Half of LTBI cases were from the nurse staff who had a low rate of compliance to treatment (only 19% of them completed the treatment). On the other hand, physicians with LTBIs had a higher compliance rate, and two-thirds of them completed the course of treatment. 

Sixty percent of LTBI cases reported working in the hospitals of the Ministy of Health (MOH), and the minority of LTBI cases (13%) were employees at King Faisal Specialist Hospital & Research Centre (KFSH&RC). However, the completion rate was higher among the LTBI cases of KFSH&RC (87%) compared with LTBI cases from MOH (33%).

## Discussion

Several studies were conducted to estimate the prevalence of LTBI using the tuberculin skin test (TST) and QuantiFERON blood test. Other researchers utilized the secondary data from the existing clinical service to display the number of LTBI-discovered cases in routine screening. The present study was designed to assess the health care workers' knowledge and perceptions toward LTBI in addition to estimating the prevalence of LTBI cases, LTBI treatment rate, and completion rate. This study strived to collect the data of the predetermined variables from the HCWs themselves in order to address their misconceptions and barriers to treatment completion.

LTBI is a preventable disease with different clinical characteristics from active TB. The current study found that the majority of participants had good knowledge regarding LTBI, including clinical features, infectivity, treatment regimens, and effectivity of the BCG vaccine. However, a considerable percentage of them were confused between LTBI and active TB regarding clinical presentation and infectivity. In a previous study [12], this finding was a challenging point in completing the treatment of LTBI because they considered the LTBI treatment unnecessary as long as there were no clinical complaints or chance of spreading the infection to others.

Classically, TB screening for HCWs has been performed using the tuberculin skin test (TST). Recently, interferon-gamma release assays (IGRAs) are a new screening tool that has been increasingly used for LTBI screening. For confirmation, symptoms screening and chest radiographs are required to exclude active TB from the positive screening tests (TST or blood tests) [13]. The present study showed that most participants underwent TST, and about a third had chest X-rays to screen and diagnose LTBI. 18% of participants in the current research had LTBI, which matches the percentage of participants who did a chest x-ray to exclude active TB.

Regarding the prevalence of latent TB, the study reported that 19% of the participants were diagnosed with latent TB disease. This figure is lower than what was previously reported for HCWs in a tertiary academic hospital in Riyadh [9] and higher than the reported prevalence of 10.8% from a cross-sectional study targeting HCWs in 2018 [14]. The screening tools used in the methodology might explain this variation in the prevalence. In the present study, we defined the latent TB case as the HCW who was previously diagnosed by a physician. 

Treatment of LTBI cases is a needed strategy to eliminate TB among HCWs who are considered at high risk of developing active TB. The current study found that 73% of participants diagnosed with LTBI accepted treatment. This low acceptance rate is in line with similar findings in previous studies [7,13]. Upon reviewing the literature in Saudi Arabia, there is no data about the treatment rate of LTBI cases in HCWs. Hence, in this study, we did not ask HCWs about their reasons for refusing LTBI treatment; it was unable to demonstrate an explanation for the low acceptance rate. However, as stated in the knowledge assessment part of the present study, the possible explanation could be poor knowledge about the difference between LTBI and active TB. Another possible explanation for this is the wrong perception regarding the low risk for progressing to TB disease among those who received the BCG vaccine earlier in life.

Among those who did accept the treatment, the overall completion rate of LTBI treatment in the present study was 55%, which is considered low compared with an American study (69%) [7] and a Korean study (73.3%) [13]. It is difficult to explain this result, but it might be related to the hospital policies, as 60% of the HCWs in the present study who did not complete the treatment reported that being the treatment optional was the reason for their low compliance. Type of treatment regimen is another factor that may help to improve compliance, 90% of participants in the present study who completed the treatment course were on the short course treatment of the rifamycin-based regimen. These results agree with the findings of other studies [7,15], in which shorter regimens for LTBI, compared to the nine-month isoniazid regimen, have been developed to increase treatment compliance.

Concerning the demographic and occupational characteristics of HCWs, the present study demonstrated the high compliance rate among LTBI cases of younger age. This finding seems to be consistent with other research [16], which found that low compliance to treatment in old age patients is mainly related to drug‐related problems as the adverse events increase with age and thus make them reluctant to undergo LTBI treatment. On the other hand, compared with other staff, the physicians in the current study had a higher compliance rate. This finding supports the idea that treatment compliance is associated with education level and the patient's perception of the disease [17]. 

There are several limitations to the present study. The present research was cross-sectional, and although the study targeted all the HCWS in Saudi Arabia, the response rate was low. Additionally, because of the limited accessibility to the national hospitals, we could not use the stratified random sampling technique - all of that influences the level of research generalizability. However, our study is the first to evaluate the acceptance and completion rates of LTBI treatment among HCWs. The current study's major strength point was assessing the accepting rate and the actual barriers to the completion of treatment from the perspective of the HCWs.

## Conclusions

This study has shown that a considerable percentage of HCWs do not know the difference between latent TB and active TB, in addition to the unsatisfactory level of acceptance and completion of the treatment of LTBI. However, the evidence from this study suggests that increasing awareness, using short LTBI treatment regimens, and changing the hospital policy regarding LTBI management will help to improve the acceptance and completion of the treatment of LTBI.

## Appendices

The questionnaire of this study was self-administered, and consisted of three following sections:

The first section includes the following demographic data: age, gender, nationality, profession, and hospital (governmental or private)

The second section includes the knowledge assessment of LTBI among health care workers by answering the following six close-ended questions:

1) What are the main symptoms that indicate latent TB infection?

- No symptoms

- Fever, cough, night sweating

2) Can latent TB infection be spread from person to person?

- Yes

- No

3) Can latent TB infection be treated with prescribed TB medicine?

- Yes

- No

4) What is the benefit of treating latent TB infection?

- To prevent active TB disease

- To treat LTBI only as it is a serious health condition.

5) How long does the treatment of latent TB infection last?

- 10 days

- 14 days

- From three months up to nine months based on the treatment regimen that planned

6) Do you think the BCG vaccine (a vaccine for TB) completely protects you from TB for your whole life?

- Yes

- No

The third section includes ten (10) questions about the medical history of latent TB as the following:

1) Had you screened against TB?

- Yes 

- No

2) Do you have this screening on annual basis? 

- Yes 

- No 

- I am not sure

3) What was the method of TB screening?

- Blood test and chest X-ray

- Skin test and chest X-ray

4) Have you been diagnosed with TB infection?  

- Yes

- No

5) Have you been diagnosed with latent TB infection?  

- Yes

- No

6) In your health facility, did your occupational health program (preventive medicine) provide you counseling or a health education session about latent TB, options of treatment course, and side effects of prescribed treatment?

- Yes

- No

7) Did you get treatment for latent TB?

- Yes

- No

- I don't have LTB

8) What is the treatment regimen that was prescribed to you?

- I don't have Latent TB

- Rifampicin for four months

- Isoniazid (INH) for nine months

- Combined (Rifampicin +Isoniazid) for three months

9) Did you complete the full course of treatment?

- Yes

- No

10) If you did not complete the treatment course, what was the reason (you can choose more than one):

- I experienced side effects of the drug

- I had a plan to complete the course but the treatment regimen was not available

- Long duration of the treatment course

- Taking the treatment in our hospital is optional
